# Association of *APOE* genotype and cerebrospinal fluid Aβ and tau biomarkers with cognitive and motor phenotype in amyotrophic lateral sclerosis

**DOI:** 10.1111/ene.16374

**Published:** 2024-06-10

**Authors:** Alessio Maranzano, Federico Verde, Antonella Dubini, Silvia Torre, Eleonora Colombo, Alberto Doretti, Francesco Gentile, Arianna Manini, Ilaria Milone, Alberto Brusati, Silvia Peverelli, Serena Santangelo, Edoardo Gioele Spinelli, Erminio Torresani, Davide Gentilini, Stefano Messina, Claudia Morelli, Barbara Poletti, Federica Agosta, Antonia Ratti, Massimo Filippi, Vincenzo Silani, Nicola Ticozzi

**Affiliations:** ^1^ Department of Neurology and Laboratory of Neuroscience IRCCS Istituto Auxologico Italiano Milan Italy; ^2^ Department of Pathophysiology and Transplantation, ‘Dino Ferrari’ Center Università degli Studi di Milano Milan Italy; ^3^ Department of Laboratory Medicine, Laboratory of Clinical Chemistry and Microbiology IRCCS Istituto Auxologico Italiano Milan Italy; ^4^ Division of Genetics and Cell Biology IRCCS San Raffaele Scientific Institute Milan Italy; ^5^ Department of Brain and Behavioural Sciences Università degli Studi di Pavia Pavia Italy; ^6^ Department of Medical Biotechnology and Molecular Medicine Università degli Studi di Milano Milan Italy; ^7^ Neurology Unit, Division of Neuroscience IRCCS San Raffaele Scientific Institute Milan Italy; ^8^ Neuroimaging Research Unit, Division of Neuroscience IRCCS San Raffaele Scientific Institute Milan Italy; ^9^ Bioinformatics and Statistical Genomics Unit IRCCS Istituto Auxologico Italiano Milan Italy; ^10^ Department of Oncology and Hemato‐Oncology Università degli Studi di Milano Milan Italy; ^11^ Vita‐Salute San Raffaele University Milan Italy; ^12^ Neurorehabilitation Unit, Division of Neuroscience IRCCS San Raffaele Scientific Institute Milan Italy; ^13^ Neurophysiology Service, Division of Neuroscience IRCCS San Raffaele Scientific Institute Milan Italy

**Keywords:** Alzheimer's disease, amyloid beta, amyotrophic lateral sclerosis, *APOE* genotype, tau proteins

## Abstract

**Objective:**

Little is known about amyotrophic lateral sclerosis (ALS)‐nonspecific cognitive deficits – most notably memory disturbance – and their biological underpinnings. We investigated the associations of the Alzheimer's disease (AD) genetic risk factor *APOE* and cerebrospinal fluid (CSF) biomarkers Aβ and tau proteins with cognitive and motor phenotype in ALS.

**Methods:**

*APOE* haplotype was determined in 281 ALS patients; for 105 of these, CSF levels of Aβ42, Aβ40, total tau (T‐tau), and phosphorylated tau (P‐tau181) were quantified by chemiluminescence enzyme immunoassay (CLEIA). The Edinburgh Cognitive and Behavioural ALS Screen (ECAS) was employed to evaluate the neuropsychological phenotype.

**Results:**

*APOE*‐E4 allele was associated with worse ECAS memory score (median, 14.0 in carriers vs. 16.0 in non‐carriers) and lower CSF Aβ42 (−0.8 vs. 0.1, log‐transformed values) and Aβ42/40 ratio (−0.1 vs. 0.3). Some 37.1% of ALS patients showed low Aβ42 levels, possibly reflecting cerebral Aβ deposition. While lower Aβ42/40 correlated with lower memory score (*β* = 0.20), Aβ42 positively correlated with both ALS‐specific (*β* = 0.24) and ALS‐nonspecific (*β* = 0.24) scores. Although Aβ42/40 negatively correlated with T‐tau (*β* = −0.29) and P‐tau181 (*β* = −0.33), we found an unexpected positive association of Aβ42 and Aβ40 with both tau proteins. Regarding motor phenotype, lower levels of Aβ species were associated with lower motor neuron (LMN) signs (Aβ40: *β* = 0.34; Aβ42: *β* = 0.22).

**Conclusions:**

*APOE* haplotype and CSF Aβ biomarkers are associated with cognitive deficits in ALS and particularly with memory impairment. This might partly reflect AD‐like pathophysiological processes, but additional ALS‐specific mechanisms could be involved.

## INTRODUCTION

Amyotrophic lateral sclerosis (ALS) is a fatal neurodegenerative disorder characterized by loss of upper (UMNs) and lower motor neurons (LMNs) and leading to progressive paralysis of voluntary muscles [1].

Increasing evidence indicates that, beyond motor symptoms, almost 50% of ALS patients show neuropsychological impairment, mainly concerning language, verbal fluency, and executive functions [2]. However, little is known about ALS‐nonspecific cognitive deficits – particularly memory disturbance – and their biological underpinnings. Since memory impairment represents the distinctive feature of Alzheimer's disease (AD) [3], some studies have investigated biological AD hallmarks in relation to cognitive symptoms in ALS patients and the possible involvement of Aβ and tau proteins in ALS pathophysiology [4]. Moreover, the acknowledged co‐occurrence of proteinopathy in neurodegenerative disorders demonstrated the importance of mixed pathology as an underrated but key element to unveil complexity behind neurodegeneration [5]. There is evidence for a potential role of amyloid precursor protein (APP) in the cellular response to axonal damage, with increased immunoreactivity of this protein in the perikarya of anterior horn cells suggesting an early protective effect [6, 7]. However, intracellular Aβ deposition might also be a late deleterious event leading to oxidative stress, activation of proapoptotic pathways [8], and TDP‐43 accumulation [9]. Concerning the other AD protein hallmarks, evaluation of the diagnostic potential of cerebrospinal fluid (CSF) total (T‐tau) and phosphorylated tau (P‐tau_181_) has provided conflicting results [10, 11]; nevertheless, T‐tau might serve as prognostic biomarker reflecting the entity of motor neuron (MN) degeneration [12] similarly to serum levels of neurofilament light chain (NFL). Recent studies have reported increased plasma phosphorylated P‐tau_181_ levels in ALS patients which were proposed as a novel marker specific to LMN degeneration [13, 14, 15]. Finally, a major risk factor for AD is represented by the E4 allele of the *APOE* gene. Whereas a pathogenic role of E4 in frontotemporal dementia (FTD) is still a matter of debate [16], with a recent article pointing out an unexpectedly increased risk in carriers of the *APOE*‐E2 allele [17], inconsistent data are available for ALS [18]. Indeed, while some evidence suggests a deleterious role of the E4 allele in ALS pathogenesis [19], other works failed to confirm an influence of APOE on clinical phenotype [20, 21].

In this work, we investigated the potential association of *APOE* haplotype, as well as CSF Aβ and tau biomarkers, with motor and cognitive/behavioral features in ALS. Specifically, we explored the incidence of amyloid and tau pathology in a deep‐phenotyped ALS cohort to estimate the occurrence of co‐pathology and to verify whether AD‐related mechanisms may be involved in ALS pathogenesis.

## METHODS

### Study cohort

A cohort of 281 Italian inpatients, diagnosed with ALS according to the El Escorial revised criteria [22], was recruited at IRCCS Istituto Auxologico Italiano between 2014 and 2022.

The following demographic and clinical data were collected: sex; age at onset; disease duration; survival; family history of ALS; motor phenotype (classic, bulbar, respiratory, flail arm, flail leg, UMN‐predominant [UMN‐p], primary lateral sclerosis [PLS], progressive muscular atrophy [PMA]) [23]; revised ALS Functional Rating Scale (ALSFRS‐R) scores at evaluation and disease progression rate (ΔFS) [24, 25]; and presence of oculomotor abnormalities as previously described [26]. Motor impairment was assessed in all patients using the following scoring systems: the Penn Upper Motor Neuron Score (PUMNS) to account for UMN regional involvement [27] and a modified version of the Lower Motor Neuron Score to account for LMN signs as previously described [28, 29]. Spinal LMN involvement was also measured using the Medical Research Council (MRC) muscle scale assessing the strength of three muscle groups for each limb (shoulder abductors, elbow flexors, wrist dorsiflexors, hip flexors, knee extensors, and ankle dorsiflexors; total score 0–60). The Edinburgh Cognitive and Behavioural ALS Screen (ECAS)‐Italian version was used to perform an extensive evaluation of both cognitive and behavioral profile of the study population [30]. Behavioral symptoms were further investigated with the Frontal Behavior Inventoy (FBI) [31]. Detailed descriptions of neuropsychological scores are provided in Supplementary Methods.

### *APOE* haplotype analysis

*APOE* haplotype was determined by imputing rs7412 and rs429358 from previously generated genotyping data [32] or by direct sequencing of *APOE* exon 4. A full description of the methodology is reported in the Supplementary Methods.

The cohort was subdivided into two groups according to the presence of at least one E4 allele. Considering the putative protective role of the E2 allele against AD and the indetermined significance of the E2|E4 genotype, patients carrying this specific genotype were excluded from analyses [33].

### CSF and serum biomarker measurement

A subcohort of patients underwent lumbar puncture as part of the diagnostic process. Measurement of CSF Aβ_42_, Aβ_40_, T‐tau, and P‐tau_181_ was performed by chemiluminescence enzyme immunoassay (CLEIA) on the Lumipulse G600II platform (Fujirebio Europe, Ghent, Belgium). An Aβ_42_/Aβ_40_ (Aβ_42/40_) ratio ≤0.069 enabled classification of patients as Aβ‐positive (A+) while an Aβ_42/40_ ratio >0.069 denoted Aβ‐negative (A–); positivity of tau pathology (T) and neurodegeneration (N) was defined by P‐tau_181_ and T‐tau levels ≥56.5 pg/mL and ≥ 404 pg/mL, respectively [34]. NFL measurement was performed on the Simoa SR‐X platform (Quanterix, Lexington, MA, USA) as previously described [35].

### Statistical analysis

Analyses were performed with statistical software R version 4.1.1. Descriptive statistics were reported as means and standard deviations for quantitative variables or frequencies (%) for categorical ones. Paired sample *t*‐test was used to compare demographic and clinical features of the whole cohort versus the CSF subcohort. Log transformation was applied to all CSF and serum biomarkers values to obtain a normal distribution, and derived measures were used in the regression analyses. Dependent and independent variables were standardized prior to regression analyses to achieve standardized beta values. Linear regression was employed for modeling the association of CSF biomarkers with motor and cognitive variables of interest, indexes of disease progression, as well as serum levels of NFL, and to investigate differences in the distribution of these variables in *APOE*‐E4 carriers and non‐carriers. Accordingly, separate comparisons between patients stratified according to amyloid and tau status were also performed. Binary logistic regression was used to assess if CSF biomarkers predicted presence or absence of cognitive impairment in different ECAS subdomains. Chi squared test was employed to compare the distribution of *APOE* genotypes in cognitively impaired and unimpaired patients. Age at evaluation was introduced as a covariate when appropriate. A sensitivity analysis was performed to assess if the association of APOE status with ECAS scores retained significance after covariation for variables known to be associated with cognitive impairment (gender, ΔFS, region of onset, and C9orf72 expansion). Survival analysis was performed with Kaplan–Meier curves and log‐rank test was used to compare survival curves across groups. Values of *p* < 0.05 were considered statistically significant.

## RESULTS

### Demographic and clinical features of ALS cohort

We studied a cohort of 281 ALS patients (179 males). Mean age at onset was 61.6 (±11.7) years, while median survival was 49.3 (41.1–57.4) months. A positive family history was reported by 41 (14.5%) patients. Site of onset was bulbar in 63 (22.4%) and spinal in 218 (77.6%) patients. Mutations in ALS‐associated genes were observed in 39 patients (21 *C9orf72*, 11 *TARDBP*, 6 *SOD1*, 1 *FUS*). According to the Strong revised criteria [36], 75 (26.7%) patients had cognitive impairment, 52 (18.5%) had behavioral impairment, and 35 (12.4%) had both cognitive and behavioral impairment, while 119 (42.3%) were cognitively and behaviorally unimpaired. Measurement of CSF biomarkers was available for 105 ALS patients (70 males) (Table 1).

**TABLE 1:** Comparison of demographic and clinical features between the whole cohort and the cerebrospinal fluid subcohort.

Feature	Total ALS cohort	CSF subcohort	*P* value
Patients, *n*	281	105	
Age at onset, mean ± SD	61.6 ± 11.7	62.8 ± 10.6	0.428
Sex, *n* (%)			
M	179 (63.7)	70 (66.7)	0.672
F	102 (36.3)	35 (33.3)	0.672
Family history, *n* (%)			
SALS	240 (85.4)	91 (86.7)	0.90
FALS	41 (14.6)	14 (13.3)	0.90
Disease duration, mean ± SD	16.5 ± 13.9	14.1 ± 10.6	0.258
Site of onset, *n* (%)			
Bulbar	63 (22.4)	25 (23.8)	0.891
Spinal	218 (77.6)	80 (76.2)	0.892
ALSFRS‐R, mean ± SD	39.8 ± 6.7	39.5 ± 6.5	0.351
PUMNS, mean ± SD	9.4 ± 7.2	9.7 ± 7.0	0.751
MRC, score mean ± SD	52.0 ± 7.9	52.4 ± 7.5	0.614
APOE status, *n* (%)			
E2|E3	32 (11.4)	14 (13.3)	0.643
E2|E4	4 (1.4)	1 (1.0)	0.644
E3|E3	188 (66.9)	75 (71.4)	0.645
E3|E4	53 (18.9)	14 (13.3)	0.646
E4|E4	4 (1.4)	1 (1.0)	0.647
C9orf72 expansion, *n* (%)	21 (7.4)	2 (1.9)	0.266
TARDBP mutation, *n* (%)	11 (3.9)	4 (3.8)	0.005
Cognitive phenotype, *n* (%)			
ALScn	119 (42.3)	46 (44.2)	0.550
ALSci	52 (18.5)	34 (32.4)	0.551
ALSbi	35 (12.4)	15 (14.3)	0.552
ALScbi	75 (26.7)	10 (9.6)	0.553
ECAS cognitive subdomains impairment, *n* (%)			
Language	59 (21.0)	21 (20)	0.962
Fluency	56 (23.5)	20 (19.0)	0.981
Executive	66 (23.5)	27 (25.7)	0.725
Memory	41 (14.6)	18 (17.1)	0.628
Visuospatial	19 (6.7)	10 (9.5)	0.474

*Note*: Demographic and clinical features of the entire cohort and the CSF subcohort. Paired sample *t*‐test to compare differences between the two groups.

Abbreviations: ALS, amyotrophic lateral sclerosis; ALSbi, ALS behaviorally impaired; ALScbi, ALS cognitively and behaviorally impaired; ALSci, ALS cognitively impaired; ALScn, ALS cognitively normal; ALSFRS‐R, ALS Functional Rating Scale Revised; APOE, apolipoprotein E; CSF, cerebrospinal fluid; ECAS, Edinburgh Cognitive and Behavioural ALS Screen; F, female; FALS, familial amyotrophic lateral sclerosis; M, male; MRC, Medical Research Council; PUMNS, Penn Upper Motor Neuron Score; SALS, sporadic amyotrophic lateral sclerosis; SD, standard deviation.

### Association of *APOE* alleles with ALS phenotype

**FIGURE 1:**
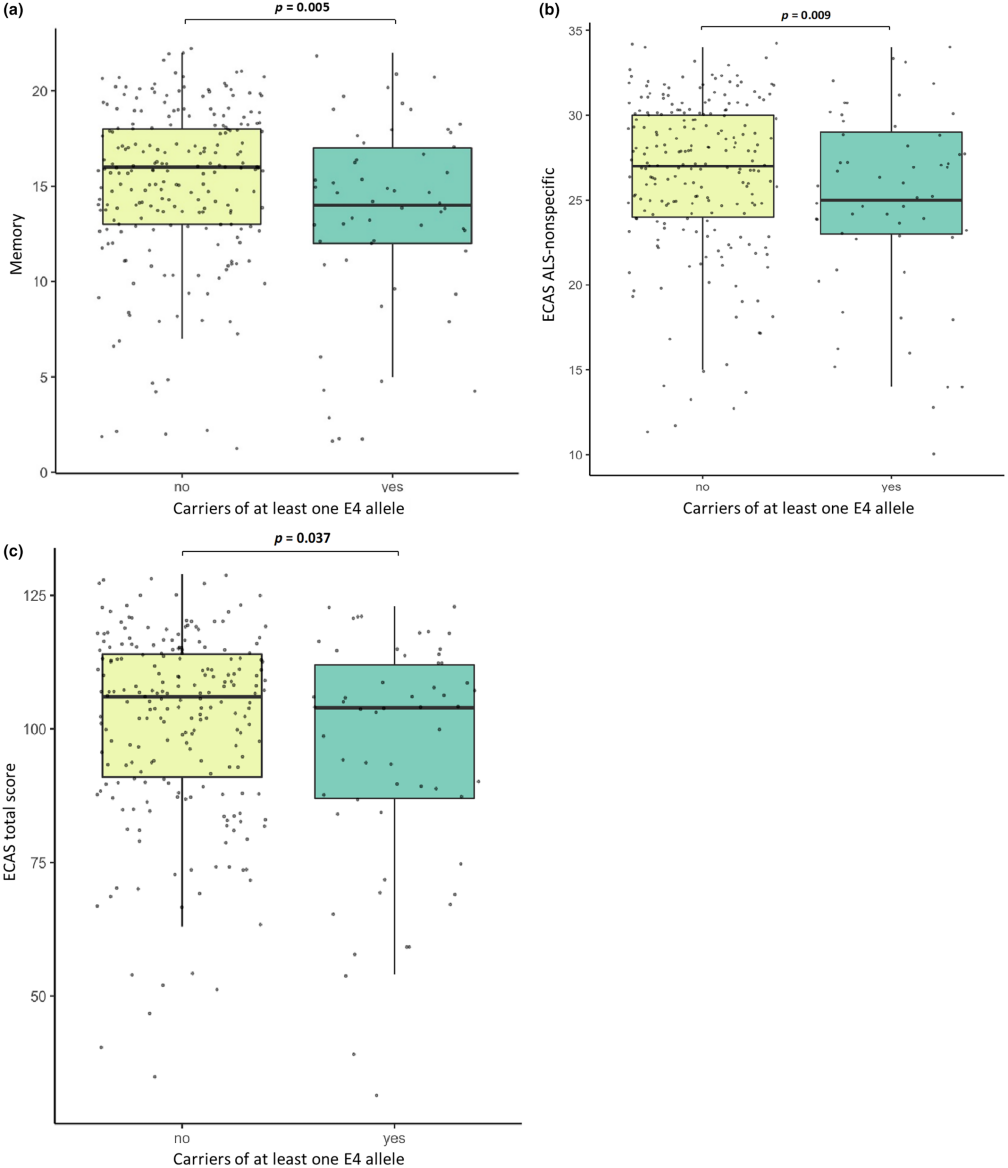
Distribution of Edinburgh Cognitive and Behavioural ALS Screen (ECAS) total (a), amyotrophic lateral sclerosis (ALS)‐nonspecific (b), and memory scores (c) in carriers and non‐carriers of at least one *APOE‐*E4 allele. For each group, the bold line shows the median, the coloured box includes the middle 50% of the values, and the extreme points of the vertical line indicate the minimum and maximum values. Black dots represent single individual scores.

In the whole cohort, genetic analysis revealed the presence of the E2|E3 genotype in 32 (11.4%), E2|E4 in 4 (1.4%), E3|E3 in 188 (66.9%), E3|E4 in 53 (18.9%), and E4|E4 in 4 (1.4%) patients. No individuals homozygous for the E2 allele were found. Allele frequencies were 6.1% for E2, 82.6% for E3, and 10.9% for E4. Regarding the CSF subcohort, the E4 allele was reported in 15/105 (14.3%) patients (14 E3|E4 and 1 E4|E4). After excluding patients with the E2|E4 genotype, individuals carrying at least one E4 allele had a lower median ECAS total score compared with non‐carriers (104.0 vs. 106.0; *p* = 0.037) indicating worse cognitive performances. This is explained by E4 carriers being more severely impaired in ALS‐nonspecific cognitive domains (25.0 vs. 27.0; *p* = 0.009), specifically memory (14.0 vs. 16.0; *p* = 0.005) (Figure 1). After stratifying for covariates that are known to influence cognitive performances (i.e., gender, site of onset, ΔFS, and C9orf72 expansion), we still observed an effect of allele E4 on ECAS total score (104.0 vs. 106.0; *p* = 0.028), ECAS ALS‐non‐specific (25.0 vs. 27.0; *p* = 0.012), and memory subdomain (14.0 vs. 16.0; *p* = 0.008). Furthermore, when considering ECAS cut‐offs, E4 carriers presented more frequently with memory impairment compared with non‐carriers [6/15 (40.0%) vs. 12/88 (13.6%); *p* = 0.036]. Conversely, the E4 allele was not associated with ALS‐specific cognitive domains, nor with behavioral symptoms assessed using both ECAS and FBI. Finally, no differences were observed regarding age at onset, site of onset, survival, and motor features.

### CSF and serum biomarkers analysis

**FIGURE 2:**
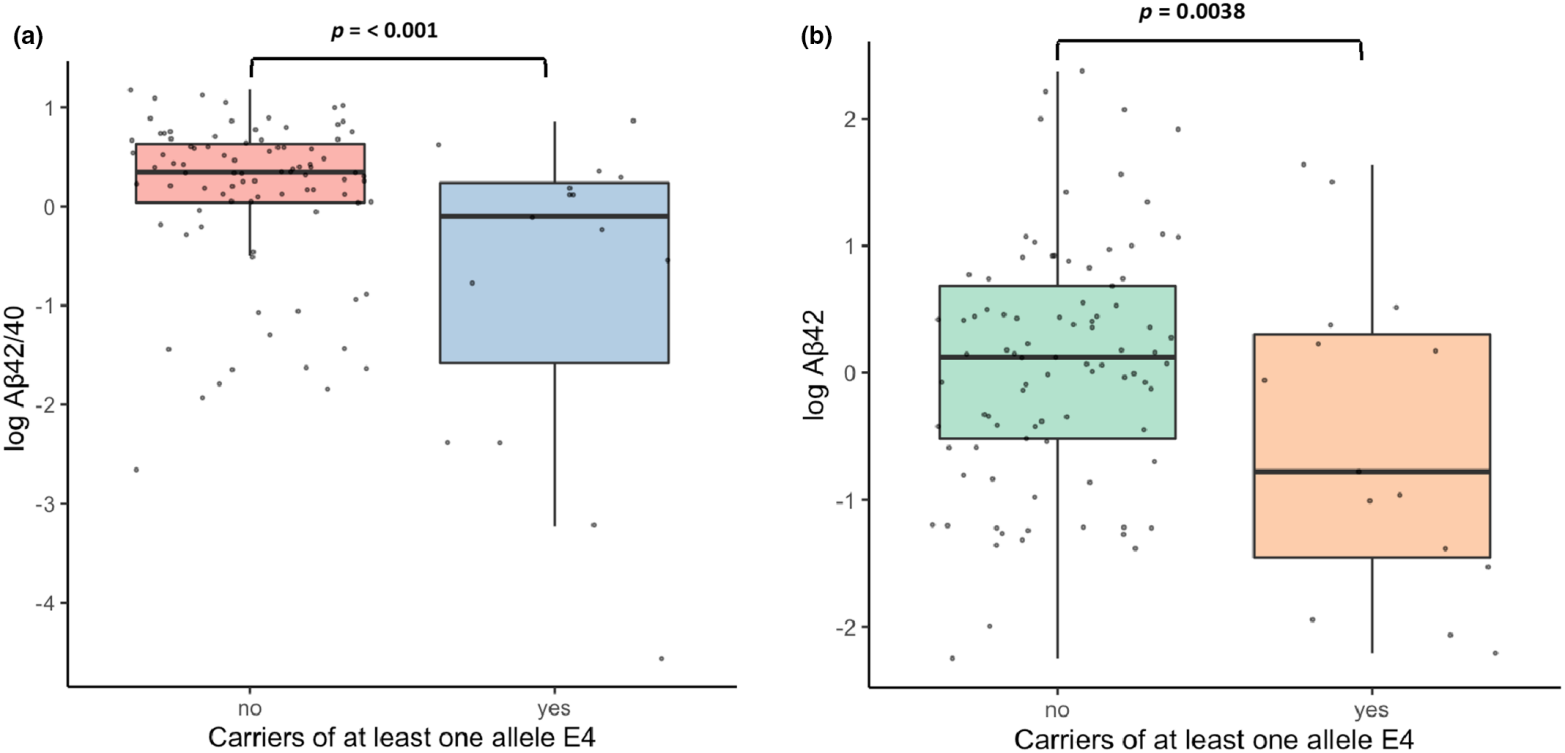
Distribution of cerebrospinal fluid (CSF) Aβ42/40 and Aβ42 levels between carriers and non‐carriers of at least one *APOE*‐E4 allele. For each group, the wide horizontal line shows the median, the coloured box includes the middle 50% of the values, and the extreme points of the vertical line show the minimum and maximum values. Black dots represent single individual scores.

Based on CSF biomarker values, 17 (16.2%) patients were classified as A+, 15 (14.2%) as T+, and 24 (22.8%) as N+, with 7 patients (6.6%) displaying an A + T + N+ profile (Table S1). Remarkably, a significant number of ALS patients (*N* = 39, 37.1%) displayed CSF Aβ_42_ levels below 599 pg/mL, that is, the cut‐off used to define A positivity when Aβ_40_ is not measured and the Aβ_42/40_ ratio cannot be calculated. The presence of at least one E4 allele was associated with significantly lower Aβ_42/40_ (−0.1 vs. 0.3; *p* < 0.001) and Aβ_42_ (−0.8 vs. 0.1; *p* = 0.038), while no association was found with other CSF biomarkers (Figure 2). Moreover, the E4 allele was more frequently observed in patients with a full AD neurochemical pattern (A + T + N+) compared with the remaining CSF cohort (4/7 [57.1%] vs. 11/97 [11.3%]; *p* = 0.005).

**FIGURE 3:**
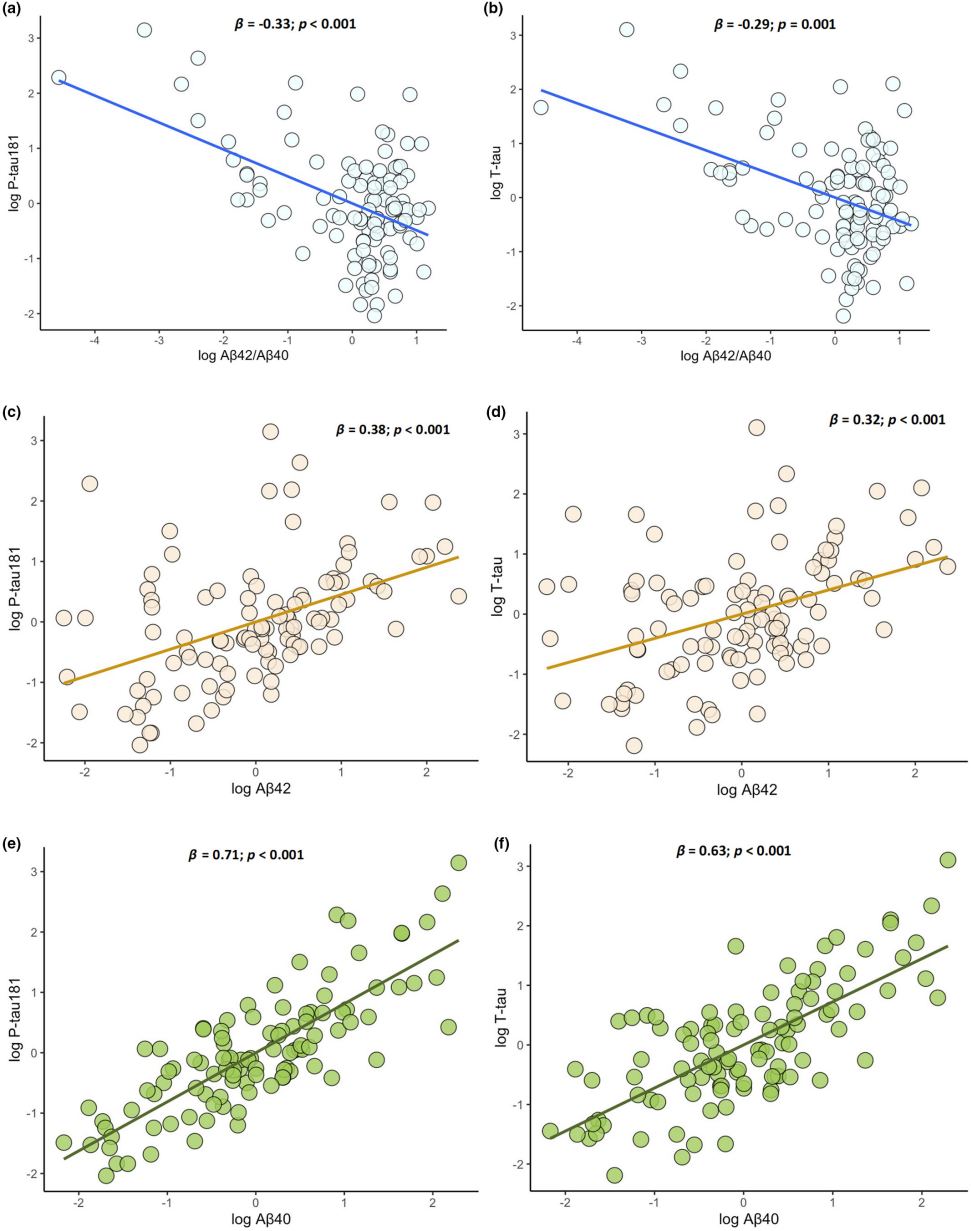
Simple dispersion with adjustment curve displaying significant negative correlations of Aβ_42/40_ ratio with T‐tau and P‐tau_181_ (a,b) and significant positive correlations of cerebrospinal fluid (CSF) Aβ_42_ (c,d) and Aβ_40_ (e,f) with T‐tau and P‐tau_181_.

As for the relationship among CSF biomarkers, lower values of Aβ_42/40_ correlated with higher levels of both P‐tau_181_ (*β* = −0.33; 95% CI = –0.49, −0.17; *p* < 0.001) and T‐tau (*β* = −0.29; 95% CI = –0.47, −0.12; *p* = 0.001) (Figure 3a,b). Moreover, A+ individuals displayed significantly higher levels of both T‐tau and P‐tau_181_ compared with A– ones (for T‐tau: 0.54 vs. −0.25; *p* = 0.002; for P‐tau_181_: 0.79 vs. −0.25; *p* < 0.001), thus reproducing the pattern observed in AD. Conversely, we found that both Aβ_42_ and Aβ_40_, when considered individually, positively correlated with P‐tau_181_ (Aβ_42_: *β* = 0.38; 95% CI = 0.22, 0.52; *p* < 0.001; Aβ_40_: *β* = 0.71; 95% CI = 0.59, 0.83; *p* < 0.001) (Figure 3c–e) and T‐tau (Aβ_42_: *β* = 0.32; 95% CI = 0.16, 0.49; *p* < 0.001; and Aβ_40_: *β* = 0.63; 95% CI = 0.48, 0.77; *p* < 0.001) (Figure 3d–f). We did not observe any association between the presence of ALS‐associated mutations and CSF biomarkers.

NFL serum levels were available for 86 of 105 patients with CSF biomarkers. The Aβ_42/40_ was inversely associated with NFL (*β* = −0.19; 95% CI = –0.37, −0.02; *p* = 0.027) while T‐tau displayed a positive association (*β* = 0.23; 95% CI = 0.04, 0.43; *p* = 0.015). No association was observed between the others CSF biomarkers and NFL.

### Association of CSF biomarkers with neuropsychological domains explored by ECAS

Lower Aβ_42/40_ correlated with lower ECAS memory scores (*β* = 0.20; 95% CI = 0.003, 0.39; *p* = 0.044), while no correlation was observed with ALS‐specific cognitive functions. When applying ECAS cut‐off values for individual cognitive domains, we again observed lower Aβ_42/40_ in patients with memory impairment compared with unimpaired ones (−0.82 vs. 0.34; *p* = 0.006).

Conversely, Aβ_42_ showed a positive correlation with both specific and nonspecific ECAS cognitive domains: total score (*β* = 0.27; 95% CI = 0.10, 0.43; *p* = 0.001), ALS‐nonspecific (*β* = 0.24; 95% CI = 0.06, 0.42; *p* = 0.008), ALS‐specific (*β* = 0.24; 95% CI = 0.08, 0.41; *p* = 0.004), memory (*β* = 0.22; 95% CI = 0.03, 0.40; *p* = 0.018), and fluency (*β* = 0.22; 95% CI = 0.04, 0.41; *p* = 0.016). These relationships held true also when using ECAS cut‐offs for individual domains, with ALS patients with verbal fluency or memory impairment displaying lower Aβ_42_ levels compared with unimpaired ones (verbal fluency: −0.36 vs. 0.17; *p* = 0.016; memory: −0.05 vs. 0.12; *p* = 0.022). Interestingly, after splitting the cohort according to the Aβ_42_ cut‐off, we again observed that patients with lower Aβ_42_ levels obtained significantly lower ECAS total (102.0 vs. 104.0; *p* = 0.025), ALS‐specific (75.0 vs. 79.0; *p* = 0.032), and fluency scores (16.0 vs. 18.0; *p* = 0.015) compared with those with normal Aβ_42_ (Figure S1).

The association between Aβ species and cognitive performance was also confirmed by the observation that lower CSF Aβ_40_ levels were positively correlated with ECAS total (*β* = 0.26; 95% CI = 0.07, 0.44; *p* = 0.005), ALS‐specific (*β* = 0.25; 95% CI = 0.07, 0.43; *p* = 0.007), and fluency scores (*β* = 0.36; 95% CI = 0.17, 0.56; *p* < 0.001), and were more frequently observed in ALS individuals with fluency impairment compared with the remaining cohort (−0.43 vs. 0.20; *p* = 0.002).

Surprisingly, we found that both CSF T‐tau and P‐tau_181_ positively correlated with ECAS fluency score (T‐tau: *β* = 0.30; 95% CI = 0.10, 0.51; *p* = 0.003; P‐tau_181_: *β* = 0.32; 95% CI = 0.11, 0.54; *p* = 0.003), and that lower levels of both tau proteins were observed in individuals with pathological fluency scores compared with those with normal scores (P‐tau_181_: −0.36 vs. 0.02; *p* = 0.005; T‐tau: −0.47 vs. 0.05; *p* = 0.005). Nevertheless, after adding Aβ_40_ as covariate in the regression analysis, the actual determinant of fluency score was Aβ_40_ (model with T‐tau: *β* = 0.30; 95% CI = 0.04, 0.55; *p* = 0.022; model with P‐tau_181_: *β* = 0.30; 95% CI = 0.02, 0.63; *p* = 0.036), while T‐tau and P‐tau_181_ lost statistical significance. Conversely, no differences in cognitive scores were observed between T+ and T– and between N+ and N– groups. (Table S2) reports correlations between CSF biomarkers and ECAS cognitive domains. Finally, we did not observe any correlations between CSF biomarkers and behavioral domains.

### Association of CSF biomarkers with motor features

**FIGURE 4:**
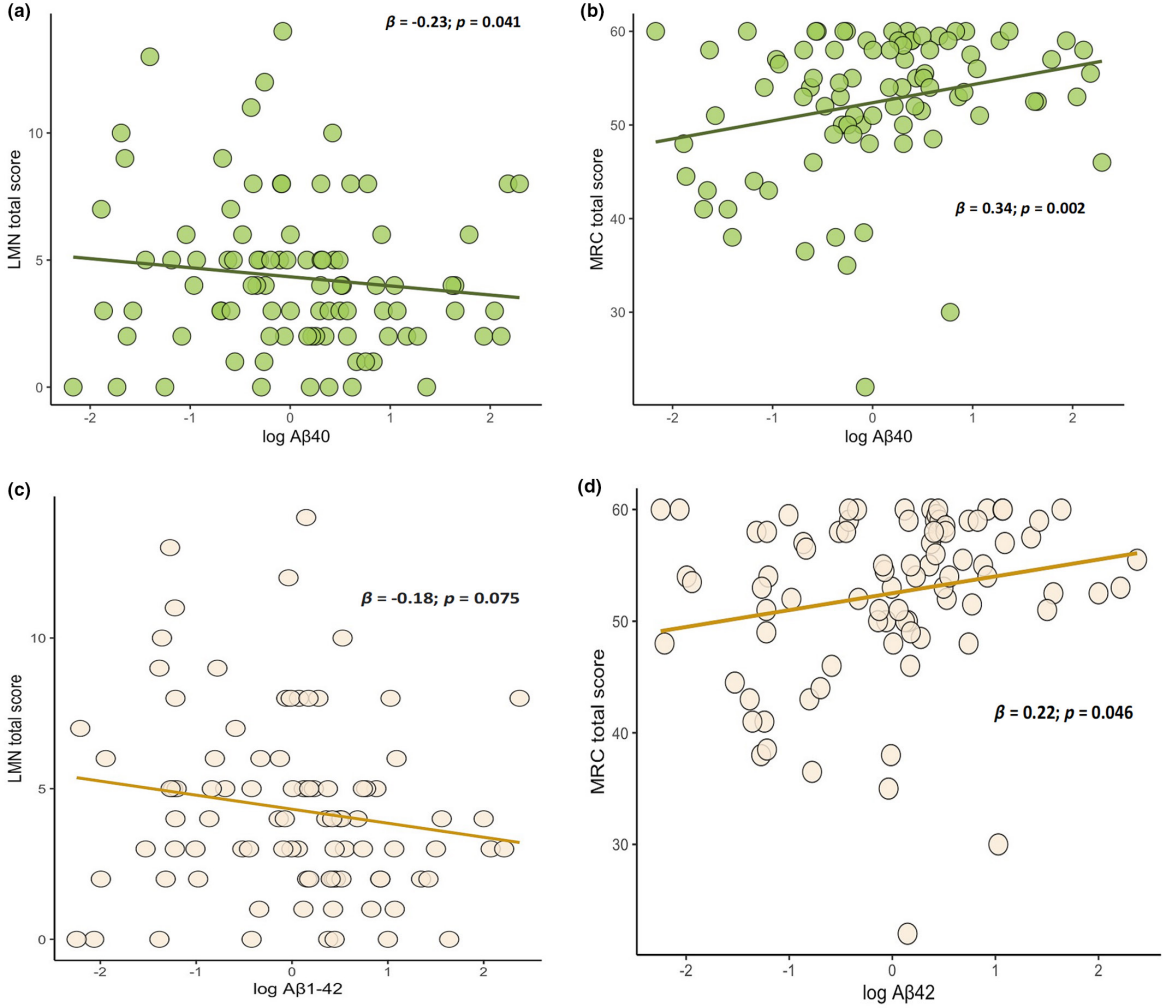
Simple dispersion with adjustment curve illustrating correlations of Aβ_40_ and Aβ_42_ with clinical indexes of lower motor neuron (LMN) impairment, namely LMN (a–c) and Medical Research Council (MRC) scores (b–d), respectively.

**FIGURE 5:**
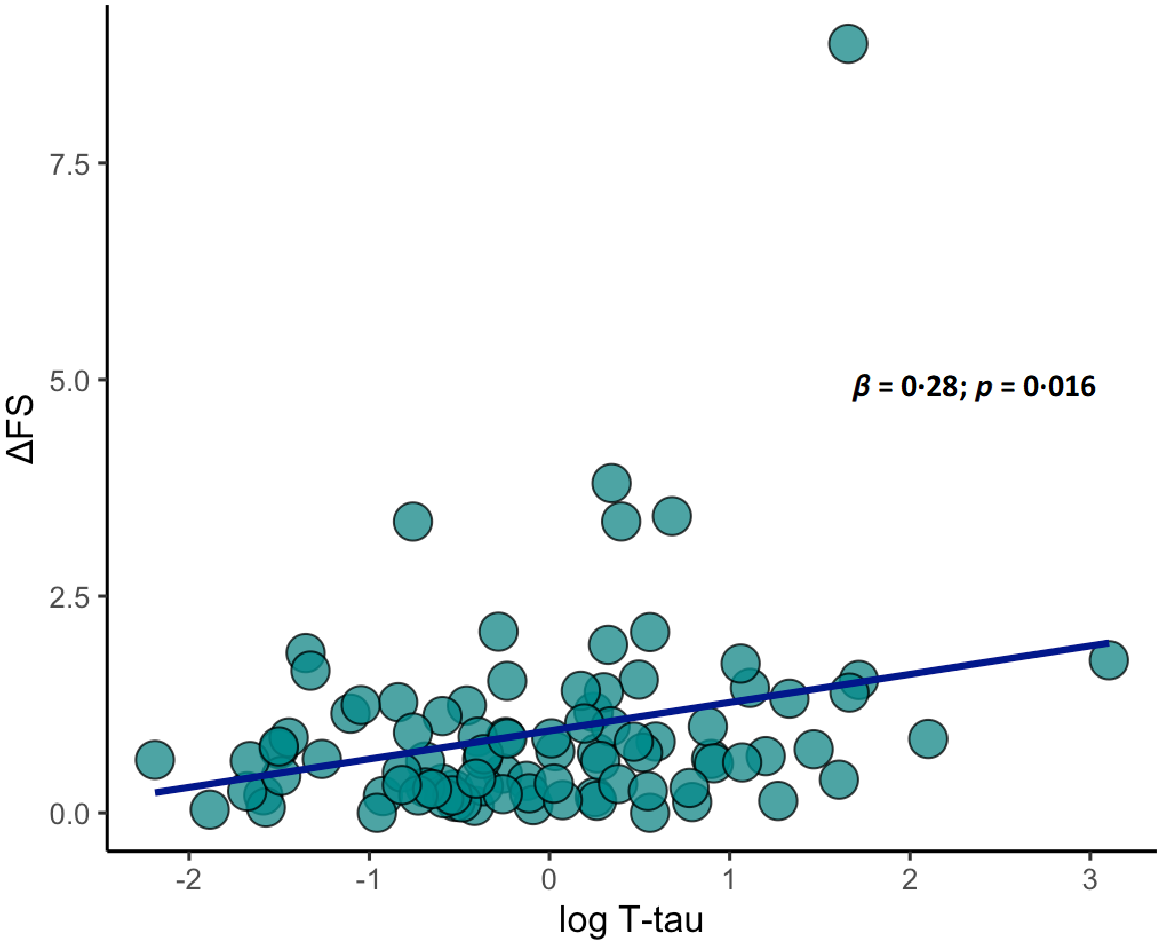
Simple dispersion with adjustment curve illustrating significant positive correlation between cerebrospinal fluid (CSF) T‐tau levels and disease progression rate (ΔFS).

While Aβ_42/40_ was not associated with motor features, we found positive correlations between both Aβ_42_ and Aβ_40_ and MRC score (Aβ_42_: *β* = 0.22; *p* = 0.046; Aβ_40_: *β* = 0.34; *p* = 0.002) and a negative correlation between Aβ_40_ and LMN score (*β* = −0.23; *p* = 0.041), indicating that CSF amyloid β species are related to the severity of LMN degeneration (Figure 4). Moreover, higher Aβ_42_ levels characterized patients with bulbar phenotype compared with those with classic ALS (0.5 vs. −0.01; *p* = 0.007) (Figure S2), whereas T‐tau positively correlated with ΔFS (*β* = 0.28; *p* = 0.016) (Figure 5).

Conversely, no association of T‐tau and P‐tau_181_ with motor phenotype and burden of UMN and LMN signs was observed, nor were CSF biomarkers associated with survival.

## DISCUSSION

Our results indicate that *APOE* genotype and CSF Aβ and tau biomarkers are associated with cognitive and motor features in ALS. Specifically, presence of at least one E4 allele and lower Aβ_42/40_ were associated with memory impairment while lower Aβ_42_ and Aβ_40_ levels were associated with diffuse cognitive deficits involving ALS‐specific functions. Lower Aβ_42_ and Aβ_40_ levels were observed in cases with more severe LMN involvement, while CSF T‐tau positively correlated with ΔFS and NFL serum levels. Classic ALS was characterized by lower Aβ_42_ levels compared with bulbar phenotype. When analyzing relationships between *APOE* and CSF biomarkers, we found associations partly explainable according to an AD‐like pathophysiological model. Indeed, presence of at least one E4 allele was associated with lower Aβ_42_ and Aβ_42/40_ values. Furthermore, Aβ_42/40_ negatively correlated with both P‐tau_181_ and T‐tau. Contrary to what is observed in AD, higher Aβ_42_ and Aβ_40_ levels were associated with higher values of both tau proteins.

Although a major ALS pathomechanism is represented by accumulation of TDP‐43 protein within MNs [37], other, less investigated biological processes might be at play. Indeed, neuropathological studies identified Aβ pathology in up to one‐half of autopsied ALS cases [38, 39]. Although this could be partly explained by the frequent occurrence of Aβ pathology in the elderly, in our cohort we found an unexpected prevalence of patients with low Aβ_42_ levels, namely twice as high as that reported for cognitively unimpaired individuals of similar age by a meta‐analysis [40], thus possibly reflecting increased cortical Aβ burden of potential pathogenic relevance. Interestingly, lower Aβ_42/40_ was associated with worse memory scores, while Aβ_42_ inversely correlated with scores in both ALS‐specific and ‐nonspecific domains. Furthermore, the presence of at least one *APOE‐*E4 allele was associated with lower values of both Aβ_42/40_ and Aβ_42_ and with more severe cognitive impairment in ALS‐nonspecific domains, particularly memory, supporting the role of *APOE* as major genetic determinant of cognitive impairment through Aβ‐dependent mechanisms. Interestingly, association of APOE haplotypes with ALS‐nonspecific domain of ECAS remained significant after covariation for variables influencing the cognitive profile. These findings are consistent with dynamics partly recapitulating AD pathophysiological processes, with Aβ deposition triggering tau accumulation as indicated by the inverse association between lower Aβ_42/40_ and tau protein levels. Therefore, Aβ pathology might contribute to cognitive impairment in ALS through AD‐like mechanisms. Morevoer, it cannot be completely ruled out that the observed effect of amyloid species on cognitive impairment in ALS could be driven by an AD co‐pathology that could be more frequent than generally considered [39, 41].

**FIGURE 6:**
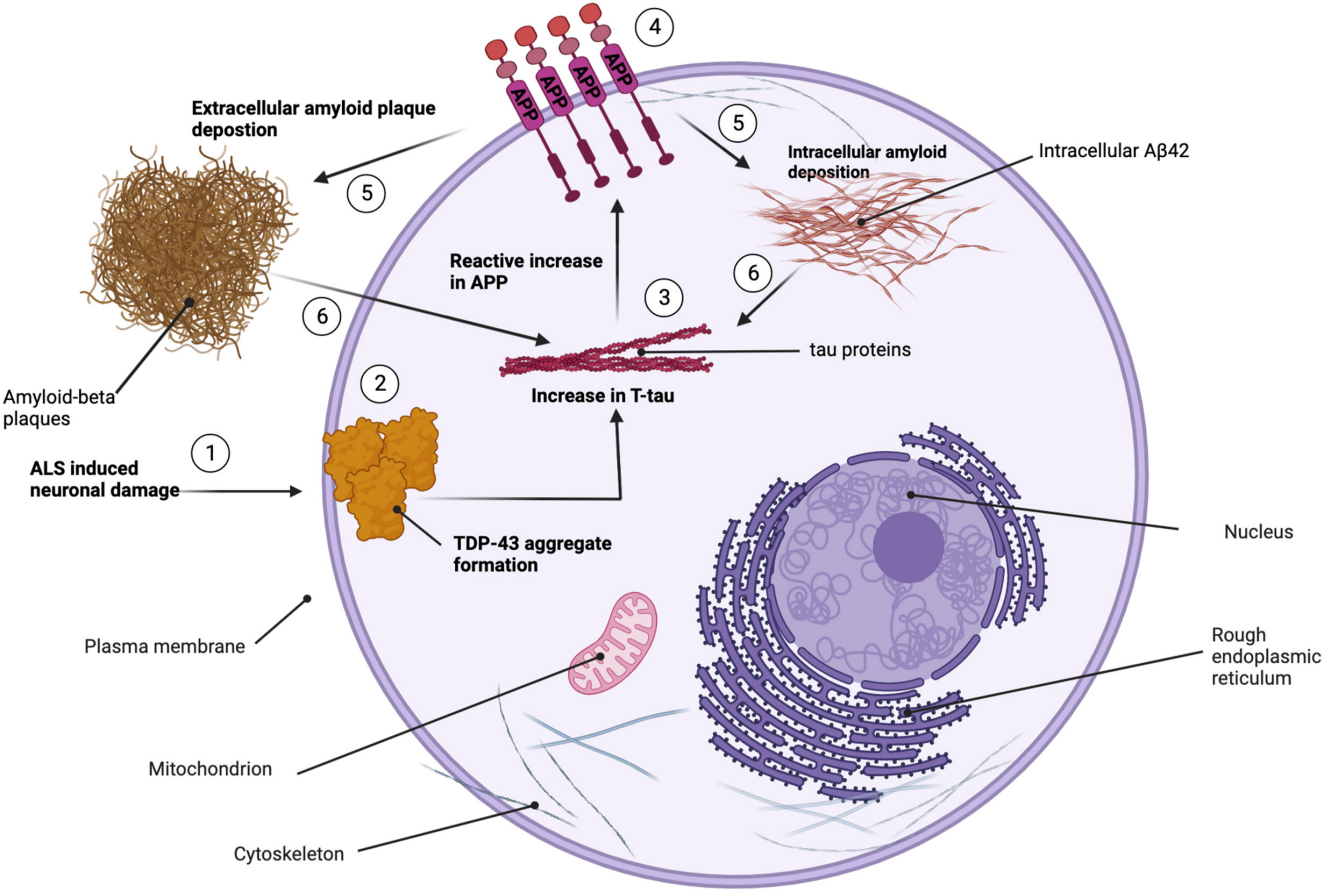
Illustration of proposed biological interplay between classic amyotrophic lateral sclerosis (ALS)‐related mechanisms of damage and Aβ pathways. Neuronal damage caused by ALS (1) resulting in TDP‐43 accumulation (2) is associated with progressive increase of T‐tau protein (3). These cellular changes lead to an indirect increase of amyloid precursor protein (APP) (protein dysmetabolism or reactive increase) (4). The APP increase may subsequently cause intracellular amyloid accumulation or extracellular amyloid plaque deposition (5), circularly triggering further neuronal damage (6). Image created with BioRender. Adopted from “Structural overview of an animal cell template” by BioRender.com (2022). Retrieved from https://app.biorender.com/biorender‐templates.

However, *APOE* could also contribute to cognitive abnormalities in ALS via different pathways, as suggested by the association between the E4 allele, TDP‐43 pathology, and hippocampal sclerosis reported in a large neuropathological study [42]. Furthermore, a comprehensive analysis of our findings suggests that strictly applying the same biological paradigms used in AD to ALS might not be totally appropriate, with the risk of overlooking relevant biological clues. Indeed, contrary to AD, in our ALS cohort we found a positive correlation between CSF levels of Aβ and tau proteins. This only applied to single Aβ species and not to the Aβ_42/40_ ratio. Therefore, it could be hypothesized that during the disease process, neuronal damage – reflected by increased T‐tau, which in turn is associated with the index of disease progression ΔFS as well as serum levels of NFL – leads to an indirect increase of APP, and subsequently of Aβ_42_ and Aβ_40_ species, as a result of impaired axoplasmic transport or reactively enhanced APP synthesis [6, 43]. In fact, APP has been reported as a marker of axonal damage across different neurological conditions, including traumatic brain injury [44]. However, it is unclear whether the putative increase in APP following neuronal damage can be explained by impairment of cell structures involved in APP metabolism such as the Golgi apparatus, which is disrupted in ALS MNs [45], or rather represents a protective mechanism against glutamate excitotoxicity or proteasomal stress [46]. Regardless, the finding that lower Aβ_42_ and Aβ_40_ levels correlated with LMN involvement is consistent with the hypothesis that APP and its fragments might be part of a protective system whose deficiency accelerates disease progression [7]. Conversely, considering the unexpectedly high prevalence of low CSF Aβ_42_ in our cohort, it could be speculated that a reactive increase in Aβ species following MN damage might favor pathological Aβ accumulation, thus leading to additional neurotoxicity. Finally, our study shows that Aβ_42_ levels are higher in patients with bulbar compared with classic phenotype. This finding suggests that a more diffuse disease process enhances Aβ accumulation, thus determining lower CSF Aβ_42_ levels. Interestingly, a previous study found lower levels of soluble APP fragments sAPPα and sAPPβ in limb‐onset compared with bulbar‐onset ALS patients [6]. In summary, our results suggest that: (1) AD pathology might contribute to cognitive dysfunction in ALS and more specifically to memory impairment; (2) it is likely that AD‐related mechanisms do not fully explain the role of Aβ species in ALS; (3) increased Aβ production might represent either a protective mechanism against neuronal damage or a direct consequence of impaired Aβ metabolism due to ALS pathological processes [47]; (4) Aβ increase may trigger its intracellular accumulation or extracellular plaque formation, thus promoting neuronal death (Figure 6); and (5) CSF T‐tau levels may represent a biomarker of disease progression in ALS.

The collateral finding that higher levels of tau proteins are associated with higher verbal fluency scores remains unclear. However, considering that the strongest association with fluency score was observed for Aβ_40_ levels and not for tau proteins, we cannot exclude that Aβ_40_ increase following early neuronal damage may elicit a protective response which initially succeeds in preserving cognitive functions before being overwhelmed by the disease.

Our study has some limitations. First, data were derived from a referral centre which is more susceptible to biases, such as longer median survival, compared with a registry population. Secondly, ECAS is tailored to assess primarily ALS‐specific cognitive deficits, and may not represent the ideal tool to assess the presence of subclinical memory deficit or other cognitive features typical of AD. Moreover, although the measurement of CSF AD biomarkers complements the characterization of cognitive and motor symptoms in our ALS cohort, it does not allow a thorough exploration of the biological pathways through which Aβ metabolism might contribute to ALS pathophysiology. In particular, it is not possible to establish whether low CSF levels of Aβ_42_ are associated with extracellular amyloid plaque deposition as described in AD or with intracellular Aβ accumulation as suggested by a previous neuropathological study [8]. Moreover, sAPPα and sAPPβ were not investigated. The lack of neuroimaging data prevented us from investigating whether amyloid and tau pathology were associated with atrophy of brain regions usually involved in AD, namely hippocampus and precuneus. Finally, despite the high concordance between CSF Aβ_42/40_ levels and amyloid‐positron emission tomography (PET) markers [48, 49], amyloid‐PET derived data would be useful to have an additional confirmation of our findings and to further investigate this topic also in relation to spatial and regional distribution.

Our findings suggest that CSF levels of Aβ and tau proteins might be associated with cognitive and motor features of ALS patients. Moreover, albeit preliminary, our results indicate that Aβ species might play a more important role in ALS pathogenesis than previously thought. Further studies, using PET‐derived data or directly neuropathological findings, are thus warranted to investigate the biological significance of these proteins in the pathological processes leading to ALS‐related neurodegeneration.

## AUTHOR CONTRIBUTIONS

**Alessio Maranzano:** Conceptualization; investigation; methodology; writing – review and editing; software; formal analysis; data curation; writing – original draft. **Federico Verde:** Conceptualization; investigation; methodology; data curation; supervision; writing – review and editing; funding acquisition. **Antonella Dubini:** Data curation; resources. **Silvia Torre:** Data curation; resources. **Eleonora Colombo:** Writing – review and editing. **Alberto Doretti:** Writing – review and editing. **Francesco Gentile:** Writing – review and editing; formal analysis. **Arianna Manini:** Writing – review and editing; formal analysis. **Ilaria Milone:** Data curation; resources. **Alberto Brusati:** Data curation; formal analysis. **Silvia Peverelli:** Data curation. **Serena Santangelo:** Writing – review and editing. **Edoardo Gioele Spinelli:** Writing – review and editing. **Erminio Torresani:** Writing – review and editing. **Davide Gentilini:** Writing – review and editing. **Stefano Messina:** Writing – review and editing. **Claudia Morelli:** Writing – review and editing. **Barbara Poletti:** Writing – review and editing; data curation; supervision; resources. **Federica Agosta:** Writing – review and editing. **Antonia Ratti:** Data curation; resources; writing – review and editing. **Massimo Filippi:** Writing – review and editing. **Vincenzo Silani:** Writing – review and editing. **Nicola Ticozzi:** Conceptualization; investigation; funding acquisition; writing – review and editing; methodology; formal analysis; supervision.

## FUNDING INFORMATION

This work was funded by BIBLIOSAN. The project was financially supported by the Italian Ministry of Health (RF‐2021‐12374238 and Ricerca Corrente to Istituto Auxologico Italiano) and AriSLA‐Fondazione Italiana di Ricerca per la SLA (Grant Azygos 2.0).

## CONFLICT OF INTEREST STATEMENT

Alessio Maranzano, Federico Verde, Antonella Dubini, Silvia Torre, Eleonora Colombo, Alberto Doretti, Francesco Gentile, Arianna Manini, Ilaria Milone, Alberto Brusati, Silvia Peverelli, Serena Santangelo, Erminio Torresani, Davide Gentilini, Stefano Messina, Claudia Morelli, Barbara Poletti, and Antonia Ratti report no disclosures. Federica Agosta is Section Editor of *NeuroImage: Clinical*; has received speaker honoraria from Biogen Idec, Roche, Zambon, and Italfarmaco; and receives or has received research supports from the Italian Ministry of Health, AriSLA (Fondazione Italiana di Ricerca per la SLA), the European Research Council, and the Foundation Research on Alzheimer Disease. Massimo Filippi is Editor in‐Chief of the *Journal of Neurology*, Associate Editor of *Human Brain Mapping*, Associate Editor of *Radiology*, and Associate Editor of *Neurological Sciences*; received compensation for consulting services from Alexion, Almirall, Biogen, Merck, Novartis, Roche, and Sanofi; speaking activities from Bayer, Biogen, Celgene, Chiesi Italia SpA, Eli Lilly, Genzyme, Janssen, Merck‐Serono, Neopharmed Gentili, Novartis, Novo Nordisk, Roche, Sanofi, Takeda, and TEVA; participation in advisory boards for Alexion, Biogen, Bristol‐Myers Squibb, Merck, Novartis, Roche, Sanofi, Sanofi‐Aventis, Sanofi‐Genzyme, and Takeda; scientific direction of educational events for Biogen, Merck, Roche, Celgene, Bristol‐Myers Squibb, Lilly, Novartis, and Sanofi‐Genzyme; he receives research support from Biogen Idec, Merck‐Serono, Novartis, Roche, Italian Ministry of Health, Fondazione Italiana Sclerosi Multipla, and ARiSLA (Fondazione Italiana di Ricerca per la SLA). Vincenzo Silani received compensation for consulting services and/or speaking activities from AveXis, Cytokinetics, Italfarmaco, LiquidWeb Srl, and Novartis Pharma AG. He receives or he has received research support from the Italian Ministry of Health, AriSla, and E‐Rare Joint Translational Call. He is on the Editorial Board of *Amyotrophic Lateral Sclerosis and Frontotemproal Degeneration, European Neurology, American Journal of Neurodegenerative Disease* and *Frontiers in Neurology*. Nicola Ticozzi received compensation for consulting services from Amylyx Pharmaceutical and Zambon Biotech SA. He received research funding from the Italian Ministry of Health and AriSLA. He is Associate Editor of *Frontiers in Aging Neuroscience*.

## Supporting information


**Data S1.** Supporting Information.

## Data Availability

Anonymized data have been published on Zenodo (10.5281/zenodo.7148972) and are available upon reasonable request from the corresponding author.
