# Supplementary material for: Association of APOE genotype and cerebrospinal fluid Aβ and tau biomarkers with cognitive and motor phenotype in amyotrophic lateral sclerosis
Source: Eur J Neurol. 2024 Jun 10;31(9):e16374. doi: 10.1111/ene.16374 (PMC11295165; doi:10.1111/ene.16374)
Supplement: Supplementary file 1 — Data S1. Supporting Information. [file ENE-31-e16374-s001.docx]

**Supplementary Appendix**

**Table of content:**

**Supplementary Method:**

Neuropsychological evaluation **………………………………………………………. 2**

Genetic analysis **...….…………………………………………………………………. 3**

**Supplementary Table 1 ………………………………………………………………….. 4**

**Supplementary Table 2 ………………………………………………………………….. 5**

**Supplementary Figure 1………………………………………………………..……….... 6**

**Supplementary Figure 2………………………………………………………..……….... 7**

**References …………………………………………………………………………………. 8**

**Supplementary Methods**

**Neuropsychological evaluation**

The ECAS total score consists of an ALS-specific subscore (assessing ALS-specific cognitive impairment, namely language, verbal fluency, and executive functions) and an ALS non-specific one (assessing memory and visuospatial domains). Each score was classified as normal vs pathologic according to validated cut-off values. ^1^ Based on the performance at ECAS, patients were classified as ALScn (cognitively normal), ALSci (cognitively impaired), ALSbi (behaviourally impaired) or ALScbi (cognitively and behaviourally impaired) according to the revised Strong criteria. ^2^ Behavioural impairment was evaluated using the ECAS Carer Interview score (range 0-10), as well as the number of behavioural symptoms recorded at the ECAS Carer Interview. Behavioural symptoms were further investigated using the Frontal Behavioral Inventory (FBI), ^3^ which consists of two subscales, exploring negative (FBI-A) and positive/disinhibited behaviours (FBI-B).

**Genetic analysis**

Human *APOE* exists as three isoforms (E2, E3, E4), defined by the two single nucleotides polymorphisms (SNPs) rs7412 and rs429358. ^4^ *APOE* haplotype was determined by imputing rs7412 and rs429358 from previously generated genotyping data ^5^ or by direct sequencing of *APOE* exon 4.

Whole-genome SNP genotyping was performed in 194 patients using the Infinium Global Screening Array (Illumina). We then performed quality control (QC) standard procedures [i.e., filtering out those samples with a SNP call rate <99%, duplicated or related with an Identity by Descend coefficient (IBD coefficient) >0.185, with an inbreeding coefficient >0.5, and with sex genotype-phenotype mismatch; removing those SNPs which had been genotyped in less than 95% of samples, which were not found at Hardy-Weinberg equilibrium, and which showed a minor allele frequency (MAF) <0.01; rejecting the samples which did not cluster into the European population according to the population structural analysis computed through the Principal Component Analysis (PCA) via Eigenstrat; removing the SNPs with allele mismatch]. The SNP imputation procedure was performed through the Michigan Imputation Server via Minimac4, by employing the European population of 1000 Genomes Phase 3 (version 5) as reference panel and the GRCh37/hg19 as array build. According to previous studies, this protocol results in high imputation accuracy of rs7412 and rs429358.^6^ After imputation, we set the R^2^ cut-off at 0.7 to remove >70% of poorly imputed SNPs at the cost of <0.5% of well-imputed SNPs, as recommended. ^7^

Direct sequencing of rs7412 and rs429358 was performed in 87 ALS patients. Genomic DNA was isolated from whole blood using Wizard Genomic DNA Purification Kit (Promega). APOE exon 4 was amplified by polymerase chain reaction (PCR) using AccuPrime GC-Rich DNA Polymerase (Invitrogen) and the following primers: FOR 5’-GCCTACAAATCGGAACTGGA-3’ and REV 5’-ACGAGGTGAAGGAGCAGGT-3’. PCR products were purified enzimatically by using illustra ExoStar (GE Healthcare) and analyzed by direct sequencing using BigDye Terminator v3.1 Cycle Sequencing Kit (Thermo Fisher Scientific) on 3500 Genetic Analyzer (Applied Biosystem).

**Supplementary Table 1: cohort stratification according to CSF amyloid and tau biomarkers and ATN classification**

| **CSF biomarker** | **Levels** | **Number** | **Percentage** |
| --- | --- | --- | --- |
| **Aβ42/40** | A- | 88 | 83.8% |
|  | A+ | 17 | 16.2% |
| **P-tau_181_** | T- | 90 | 85.7% |
|  | T+ | 15 | 14.3% |
| **T-tau** | N- | 81 | 77.1% |
|  | N+ | 24 | 22.9% |
| **ATN**  A-T-N- 72 68.6% | | | |
| A-T-N + 9 8.6% | | | |
|  | A-T+N + | 7 | 6.6% |
|  | A+T-N- | 8 | 7.6% |
|  | A+T-N+ | 1 | 0.9% |
|  | A+T+N- | 1 | 0.9% |
|  | A+T+N+ | 7 | 6.6% |

**Supplementary Table 1:** classification of ALS cohort according to CSF amyloid and tau biomarkers and ATN staging system. ^8^Abbreviation: A+ = values of CSF Aβ_42/40_ ≤ 0.069 pg/ml; A- = values of CSF Aβ_42/40_ ≥ 0.069 pg/ml; T+ = values of CSF P-tau_181_ ≥ 56.5 pg/ml; T- = values of CSF P-tau_181_ ≤ 56.5 pg/ml; N+ = values of CSF T-tau ≥ 404 pg/ml; N- = values of CSF T -tau  ≤ 404 pg/ml;

**Supplementary Table 2: correlation of CSF amyloid ant tau biomarkers with ECAS cognitive domains**

|  | **CSF biomarkers** | | | | |
| --- | --- | --- | --- | --- | --- |
| **ECAS domains** | **Aβ_42/40_** | **Aβ_42_** | **Aβ_40_** | **T-tau** | **P-tau_181_** |
| Language | *0*.*700* | *0*.*053* | *0*.*079* | *0*.*516* | *0*.*413* |
| Fluency | *0*.*189* | ***0***.***016*** | ***< 0***.***001*** | ***0***.***003*** | ***0***.***003*** |
| Executive | *0*.*104* | *0*.*072* | *0*.*422* | *0*.*934* | *0*.*810* |
| Memory | ***0.044*** | ***0*.*018*** | *0*.*130* | *0*.*526* | *0*.*987* |
| Visuospatial | *0*.*979* | *0*.*088* | *0*.*053* | *0*.*284* | *0*.*084* |
| ALS-specific | *0*.*530* | ***0***.***004*** | ***0***.***007*** | *0*.*066* | *0*.*087* |
| ALS-non-specific | *0*.*072* | ***0***.***008*** | *0*.*059* | *0*.*737* | *0*.*690* |
| ECAS total score | *0*.*310* | ***0***.***001*** | ***0***.***005*** | *0*.*149* | *0*.*123* |

**Supplementary Table 2:** p values for correlations between CSF biomarkers and main ECAS cognitive domains. ALS-specific score is calculated as the the sum of language, verbal fluency, and executive functions subdomains; ALS non-specific score is calculated as the sum of memory and visuospatial subdomains; ECAS total score is calculated as the sum of ALS-specific + ALS-non-specfic domains;

Significant correlations are displayed in bold.

**Supplementary Figure 1: association of CFS Aβ_42_ levels with cognitive phenotype**

**Supplementary Figure 1:** Distribution of ECAS ALS total (A), ALS specific (B) and Fluency scores (C) between two groups of patients stratified according to CSF reference levels of Aβ_42_ (< and > 599 pg/ml respectively). For each group, the bold line shows the median, the colored box includes the middle 50% of the data and extreme points of vertical line show the minimum and maximum values. Black dots represent single individual score.

Abbreviation: Aβ_42_ + = values of CSF Aβ_1-42_ under 599 pg/ml; Aβ_42_ - = values of CSF Aβ_42_ above or equal to 599 pg/ml; IQR= interquartile range

**Supplementary Figure 2: Association of Aβ42 with motor phenotype.**

**Supplementary Figure 2:** Distribution of CSF Aβ42 levels between patients with bulbar and classic motor phenotype. For each group, the bold line shows the median, the coloured box includes the middle 50% of the data and extreme points of vertical line show the minimum and maximum values. Black dots represent single individual scores.

**References**

1. Poletti B, Solca F, Carelli L, et al. The validation of the Italian Edinburgh Cognitive and Behavioural ALS Screen (ECAS). *Amyotrophic Lateral Sclerosis and Frontotemporal Degeneration*. 2016;17(7-8):489-498. doi:10.1080/21678421.2016.1183679

2. Strong MJ, Abrahams S, Goldstein LH, et al. Amyotrophic lateral sclerosis - frontotemporal spectrum disorder (ALS-FTSD): Revised diagnostic criteria. *Amyotrophic Lateral Sclerosis and Frontotemporal Degeneration*. 2017;18(3-4):153-174. doi:10.1080/21678421.2016.1267768

3. Alberici A, Geroldi C, Cotelli M, et al. The Frontal Behavioural Inventory (Italian version) differentiates frontotemporal lobar degeneration variants from Alzheimer’s disease. *Neurol Sci*. 2007;28(2):80-86. doi:10.1007/s10072-007-0791-3

4. Hauser P, Ryan R. Impact of Apolipoprotein E on Alzheimer’s Disease. *CAR*. 2013;10(8):809-817. doi:10.2174/15672050113109990156

5. van Rheenen W, van der Spek RAA, Bakker MK, et al. Common and rare variant association analyses in amyotrophic lateral sclerosis identify 15 risk loci with distinct genetic architectures and neuron-specific biology. *Nat Genet*. 2021;53(12):1636-1648. doi:10.1038/s41588-021-00973-1

6. Vuoksimaa E, Palviainen T, Lindgren N, Rinne JO, Kaprio J. Accuracy of Imputation for Apolipoprotein E ε Alleles in Genome-Wide Genotyping Data. *JAMA Netw Open*. 2020;3(1):e1919960. doi:10.1001/jamanetworkopen.2019.19960

7. Li Y, Abecasis GR. Mach 1.0: Rapid haplotype reconstruction and missing genotype inference.2006.

8. Jack CR, Bennett DA, Blennow K, et al. NIA‐AA Research Framework: Toward a biological definition of Alzheimer’s disease. *Alzheimer’s &amp; Dementia*. 2018;14(4):535-562. doi:10.1016/j.jalz.2018.02.018
